# Multiplex proteomics for prediction of major cardiovascular events in type 2 diabetes

**DOI:** 10.1007/s00125-018-4641-z

**Published:** 2018-05-24

**Authors:** Christoph Nowak, Axel C. Carlsson, Carl Johan Östgren, Fredrik H. Nyström, Moudud Alam, Tobias Feldreich, Johan Sundström, Juan-Jesus Carrero, Jerzy Leppert, Pär Hedberg, Egil Henriksen, Antonio C. Cordeiro, Vilmantas Giedraitis, Lars Lind, Erik Ingelsson, Tove Fall, Johan Ärnlöv

**Affiliations:** 1 Division of Family Medicine and Primary Care, Department of Neurobiology, Care Sciences and Society (NVS), Karolinska Institutet, Alfred Nobels Allé 23, SE 14183 Huddinge, Sweden; 2 Department of Medical Sciences, Uppsala University, Uppsala, Sweden; 3 Department of Medical and Health Sciences, Linköping University, Linköping, Sweden; 4 School of Technology and Business Studies/Statistics, Dalarna University, Falun, Sweden; 5 School of Health and Social Studies, Dalarna University, Falun, Sweden; 6 Department of Medical Epidemiology and Biostatistics, Karolinska Institutet, Stockholm, Sweden; 7 Centre for Clinical Research, Uppsala University, Västerås, Sweden; 8 Department of Hypertension and Nephrology, Dante Pazzanese Institute of Cardiology, São Paulo, Brazil; 9 Department of Public Health and Caring Sciences, Geriatrics, Uppsala University, Uppsala, Sweden; 10 Department of Medicine, Division of Cardiovascular Medicine, Stanford University School of Medicine, Stanford, CA USA

**Keywords:** Biomarkers, Major adverse cardiovascular event, Proteomics, Risk, Type 2 diabetes

## Abstract

**Aims/hypothesis:**

Multiplex proteomics could improve understanding and risk prediction of major adverse cardiovascular events (MACE) in type 2 diabetes. This study assessed 80 cardiovascular and inflammatory proteins for biomarker discovery and prediction of MACE in type 2 diabetes.

**Methods:**

We combined data from six prospective epidemiological studies of 30–77-year-old individuals with type 2 diabetes in whom 80 circulating proteins were measured by proximity extension assay. Multivariable-adjusted Cox regression was used in a discovery/replication design to identify biomarkers for incident MACE. We used gradient-boosted machine learning and lasso regularised Cox regression in a random 75% training subsample to assess whether adding proteins to risk factors included in the Swedish National Diabetes Register risk model would improve the prediction of MACE in the separate 25% test subsample.

**Results:**

Of 1211 adults with type 2 diabetes (32% women), 211 experienced a MACE over a mean (±SD) of 6.4 ± 2.3 years. We replicated associations (<5% false discovery rate) between risk of MACE and eight proteins: matrix metalloproteinase (MMP)-12, IL-27 subunit α (IL-27a), kidney injury molecule (KIM)-1, fibroblast growth factor (FGF)-23, protein S100-A12, TNF receptor (TNFR)-1, TNFR-2 and TNF-related apoptosis-inducing ligand receptor (TRAIL-R)2. Addition of the 80-protein assay to established risk factors improved discrimination in the separate test sample from 0.686 (95% CI 0.682, 0.689) to 0.748 (95% CI 0.746, 0.751). A sparse model of 20 added proteins achieved a C statistic of 0.747 (95% CI 0.653, 0.842) in the test sample.

**Conclusions/interpretation:**

We identified eight protein biomarkers, four of which are novel, for risk of MACE in community residents with type 2 diabetes, and found improved risk prediction by combining multiplex proteomics with an established risk model. Multiprotein arrays could be useful in identifying individuals with type 2 diabetes who are at highest risk of a cardiovascular event.

**Electronic supplementary material:**

The online version of this article (10.1007/s00125-018-4641-z) contains peer-reviewed but unedited supplementary material, which is available to authorised users.

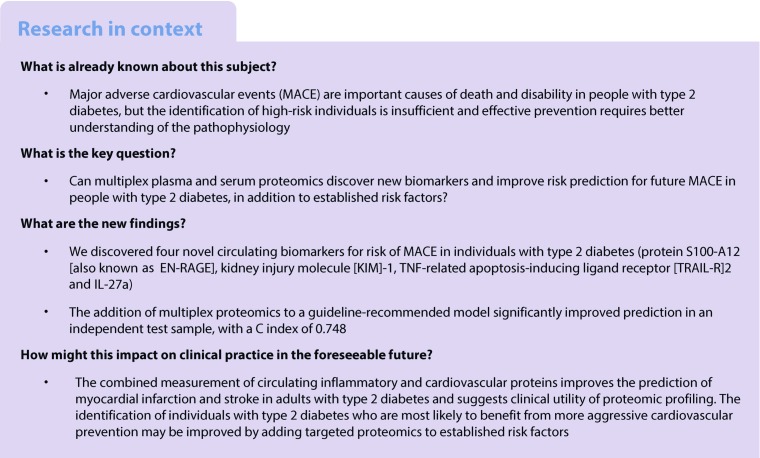



## Introduction

The prevalence of type 2 diabetes is increasing worldwide, with currently over 400 million individuals diagnosed and over 190 million undiagnosed as having diabetes [1]. Up to 40% of the US population will develop type 2 diabetes during their lifetime, and type 2 diabetes is an important contributor to major adverse cardiovascular events (MACE) such as myocardial infarction and stroke—the leading causes of morbidity and mortality in Western countries [2]. Diabetes is one of the strongest risk factors for MACE [3], and one major treatment goal in type 2 diabetes is to prevent MACE. However, compared with those without diabetes, most individuals with type 2 diabetes remain at increased risk of MACE despite optimal treatment according to current guidelines [4, 5]. Cardiovascular prevention is further complicated by increased rates of drug side effects in people with diabetes, including potential adverse glycaemic effects of lipid-modifying agents [6, 7].

Newer glucose-lowering drugs such as sodium–glucose co-transporter 2 inhibitors, and cholesterol-modifying agents such as proprotein convertase subtilisin/kexin type 9 (PCSK9) inhibitors, in addition to standard treatment reduce cardiovascular risk in high-risk individuals [8, 9]. The high treatment costs and potential side effects, however, currently prohibit their prescription in the majority of individuals with diabetes [7, 10, 11]. Identification of high-risk individuals in whom the benefits of aggressive prevention outweigh the costs and side effects is therefore crucial. Clinical decision-making based on overall cardiovascular risk in addition to individual risk factors can improve outcomes, as demonstrated for antihypertensive treatment [12]. Available risk models for MACE in type 2 diabetes are, however, only moderately accurate [13, 14], and there is a need for better prediction tools to guide healthcare.

Measuring circulating proteins with presumed roles in cardiovascular pathology by targeted proteomics is a promising approach for biomarker discovery [15]. The translation of proteomics into the clinic, however, has so far been hampered by the resource-demanding technology. Multiplex protein arrays that rely on common methods such as PCR, require small sample volumes and are available at a fraction of the cost of large-scale platforms may provide a clinically applicable method for individualised treatment based on biomarker profiles. One such technique, the proximity extension assay, has been shown to be useful for biomarker discovery in cardiometabolic disease [16–18]. Multiprotein assays have been used to discover new risk markers for cardiovascular disease in type 2 diabetes [19], but the proximity extension method has not been tested to predict risk of MACE in type 2 diabetes.

Here, we used a proximity extension assay to measure the abundance of 80 cardiovascular and inflammatory proteins in plasma and serum from six prospective community cohorts of middle-aged people (30–77 years of age) with type 2 diabetes. We aimed to identify markers of future risk of MACE and to assess the assay’s performance against an established risk model in the Swedish National Diabetes Register for the prediction of MACE.

## Methods

### Participating cohorts

#### Cardiovascular Risk Factors in Patients with Diabetes: a Prospective Study in Primary Care

The study Cardiovascular Risk Factors in Patients with Diabetes: a Prospective Study in Primary Care (CARDIPP; ClinicalTrials.gov NCT01049737) [20] recruited outpatients aged 55–65 years with type 2 diabetes from 25 primary healthcare centres in the counties of Östergötland and Jönköping, Sweden, between November 2005 and December 2008. Counties were selected to represent different demographic, rural and urban, small- and large-intake areas. Specialist diabetes nurses performed annual assessments [20]. Out of 761 consecutively enrolled participants, 708 with available outcome data and plasma samples were included in the present investigation.

#### Prospective Investigation of the Vasculature in Uppsala Seniors

In 2001, a non-selective sample of Uppsala community residents aged 70 years were recruited to participate in the longitudinal Prospective Investigation of the Vasculature in Uppsala Seniors **(**PIVUS) study [21] to evaluate measures of endothelial function; 1016 (50.2%) out of 2025 invited individuals enrolled. Follow-up biomedical assessments have been performed at 5-yearly intervals (for more information, please see www.medsci.uu.se/pivus/). All 98 participants with type 2 diabetes at baseline were included in the present study.

#### Uppsala Longitudinal Study of Adult Men

In 1970, all 2841 male residents of Uppsala county, Sweden, who had been born between 1920 and 1924 were invited to participate in the Uppsala Longitudinal Study of Adult Men (ULSAM) study [22], and 2322 (81.7%) were enrolled. Health assessments have been performed regularly since then (for details, please see www.pubcare.uu.se/ulsam/), and the current study includes all 86 participants with type 2 diabetes at an assessment age of 77 years.

#### Study of Atherosclerosis in Västmanland

Between November 2005 and May 2011, the Study of Atherosclerosis in Västmanland **(**SAVa) [23] enrolled a total of 2315 individuals into three cohorts composed of participants with acute myocardial infarction (Västmanland Myocardial Infarction Study [VaMIS]; NCT01452178), participants with peripheral artery disease (Peripheral Arterial Disease in Västmanland [PADVa]; NCT01452165) and matched control individuals (SAVa-control; for more information, please see https://savastudy.se/). The current study uses data and samples from PADVa and SAVa-control. PADVa recruited consecutive participants referred to the Vascular Ultrasound Laboratory of Västmanland County Hospital, Västerås, Sweden, who fulfilled one of three inclusion criteria: (1) at least mild internal carotid artery stenosis; (2) claudication symptoms with an ankle–brachial pressure index ≤0.90; or (3) claudication symptoms with signs of arterial occlusive disease in the ipsilateral extremity on ultrasound examination. Out of 614 eligible individuals, 452 (73.6%) enrolled. Control participants (*n* = 692) were recruited from Swedish residents in the Swedish population register who were matched by age, sex and municipality to participants enrolled in VaMIS. The current study includes all 80 individuals in SAVa-control and 99 in PADVa who were diagnosed with type 2 diabetes at baseline.

#### Malnutrition, Inflammation and Vascular Calcification cohort

The aim of the Malnutrition, Inflammation and Vascular Calcification **(**MIVC) cohort [24] is to study risk factors in kidney disease. Between March 2010 and March 2013, the study enrolled 300 consecutive outpatients who were not undergoing dialysis with stage 3–5 chronic kidney disease at the Dante Pazzanese Institute of Cardiology, São Paolo, Brazil. The current study includes all 140 participants with type 2 diabetes.

### Ethical permission

Participants provided written informed consent, and the study was conducted according to the Declaration of Helsinki. Ethical permission was granted by the ethics committees of Linköping University (Dnr. 26–05; CARDIPP), Uppsala University (Dnr. 251/90 and 97/329 for ULSAM; Dnr. 00419 and 2005/M-079 for PIVUS; Dnr. 2005:382 for SAVa/PADVa) and the Dante Pazzanese Institute of Cardiology (São Paolo, Brazil).

### Inclusion criteria and outcome definition

In CARDIPP, MIVC, SAVa-control and PADVa, type 2 diabetes was defined as a physician diagnosis of type 2 diabetes according to national guidelines (at least two separate fasting glucose levels ≥7.0 mmol/l, or at least two separate HbA_1c_ concentrations >48 mmol/mol [>6.5%; in MIVC], or prescription of diabetes medication). In ULSAM, type 2 diabetes was defined as HbA_1c_ >48 mmol/mol (>6.5%), prescription of diabetic medication or a fasting plasma glucose level ≥7.0 mmol/l. In 25 out of 86 participants included in ULSAM, diabetes was diagnosed by elevated fasting glucose alone. In PIVUS, type 2 diabetes was defined as a physician diagnosis, prescription of glucose-lowering medication or a fasting plasma glucose level ≥7.0 mmol/l. In the PIVUS group, diabetes was diagnosed by elevated fasting glucose alone in 21 out of the 98 included participants. Individuals without available fasting frozen plasma or serum samples, or with missing outcome data, were excluded. MACE was defined as a new episode of fatal or non-fatal myocardial infarction (I21 in ICD-10; www.who.int/classifications/icd/en/) or fatal/non-fatal stroke (I60–I63), whichever occurred first, and was from obtained from hospital and death register linkage.

### Covariate definitions

To adjust for established risk factors, we selected all variables included in the Swedish National Diabetes Register (NDR) calculator for 5 year risk of MACE in individuals with type 2 diabetes [13]: sex, systolic blood pressure (mmHg), BMI (kg/m^2^), current smoking, diagnosis of atrial fibrillation, history of myocardial infarction or stroke, HbA_1c_ (mmol/mol, %), HDL-cholesterol and total cholesterol (mmol/l), duration of type 2 diabetes (days), microalbuminuria (3–30 mg/mmol urinary creatinine) and macroalbuminuria (>30 mg/mmol urinary creatinine). Additional covariates included current antihypertensive, statin or diabetes medication, LDL-cholesterol (mmol/l) and eGFR (ml min^−1^ [1.73 m]^−2^), calculated with plasma creatinine according to sex, age and ethnicity). Missing values in covariates were imputed by multivariate imputation by chained equations with predictive mean matching using all other covariates and averaged across five iterations. Imputed values were compared against recorded values to assess for aberrations.

### Multiplex protein assay

Blood samples were obtained from individuals instructed to fast overnight, and were then spun down and stored as serum (ULSAM) or EDTA plasma samples (all other cohorts) at −70°C until analysis. The Proseek CVD Multiplex 96×96 (Olink, Uppsala, Sweden) measures 92 cardiovascular or inflammatory proteins and four internal control samples using the proximity extension assay method (details on quality control, validation and content of the assay are available in electronic supplementary material [ESM] Table 1 and ESM Methods). It has previously been applied to discovering biomarkers for cardiometabolic traits [16–18]. In brief, approximately 10 μl of sample were assayed on a 96-well plate, and protein abundance was measured by PCR based on the binding of two specific antibodies for each protein. Log_2_-scaled abundance values adjusted for technical variation with internal controls were transformed to a mean of zero and an SD of 1. Proteins with >15% missing values were excluded. Other missing values were imputed by the lower limit of the detection threshold divided by two. The numbers of missing values are given in ESM Table 2. A total of 12 proteins had >15% missing values in at least one cohort and were excluded, leaving 80 proteins for inclusion in the study.

### Statistical analysis

#### Design

**Fig. 1:**
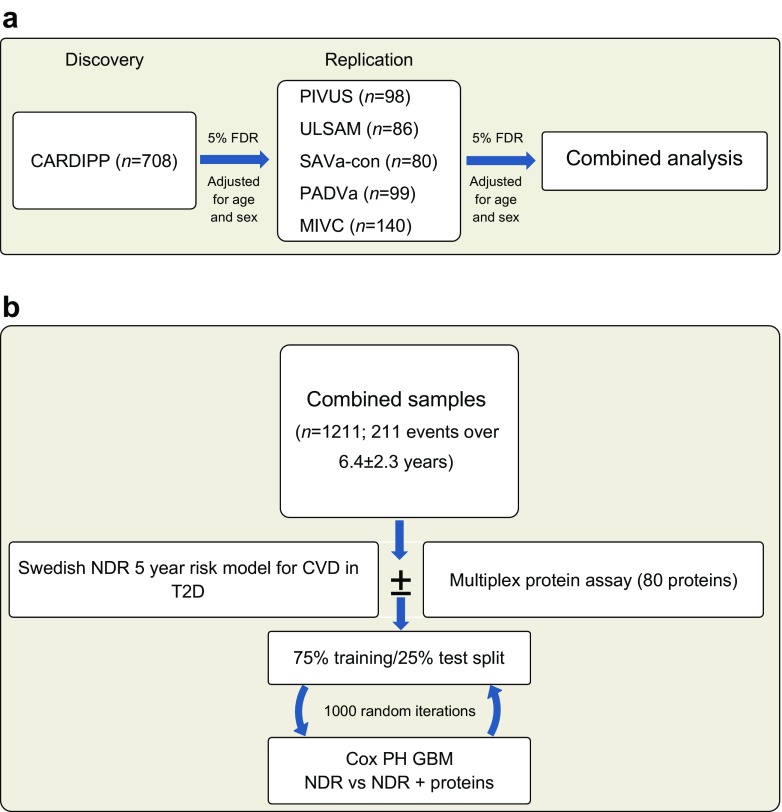
Study flowchart showing (**a**) cohorts and (**b**) further details of the analysis. The combined analysis was adjusted for: sex, current smoking, duration of type 2 diabetes (T2D), BMI, systolic BP, HbA_1c_, LDL-cholesterol, microalbuminuria, statin use, previous cardiovascular disease (CVD), atrial fibrillation and eGFR. NDR predictors were: age of onset of T2D, T2D duration, total cholesterol/HDL-cholesterol, HbA_1c_, systolic BP, BMI, sex, current smoking, microalbuminuria, macroalbuminuria, atrial fibrillation and previous CVD. PH, proportional hazards

The study was divided into two parts, one aimed at biomarker discovery and one at risk prediction (Fig. 1). In part 1, the largest sample, from CARDIPP, was used for discovery, and all other cohorts, combined at the individual level, were used for replication. In part 2, the combined discovery and replication cohorts were randomly split into a 75% training and a 25% test set to assess whether the different proteins would improve the prediction of MACE.

#### Part 1: biomarker discovery

Cox proportional hazards regression adjusted for age and sex was used for each protein, with time-to-MACE as outcome. Participants were considered to be at risk until the occurrence of MACE or until the last day of follow-up. An inverse Gaussian frailty effect was included to adjust for heterogeneity between cohorts. The linearity of associations with risk of MACE was assessed by adding a spline term to the linear model (using the pspline function in R with defaults, and retaining the linearity assumption if the regression β coefficient’s *p* value exceeded 0.05). Proportional hazards assumptions were assessed in Schoenfeld residual plots and tests of weighted residuals (threshold *p* < 0.05). The protein assay does not provide standard concentration units, and values were scaled to a mean of zero and an SD of 1. Proteins associated below a 5% false discovery rate (FDR) in the CARDIPP discovery sample were tested in the replication sample, and associations at <5% FDR at the replication stage were considered significant. To test for independent associations with MACE, we additionally adjusted for the following established cardiovascular risk factors [25] that were available in the cohorts: atrial fibrillation, BMI, HbA_1c_, LDL-cholesterol, microalbuminuria, systolic blood pressure, sex, smoking, statins, duration of type 2 diabetes, history of cardiovascular disease and eGFR.

#### Part 2: risk prediction

To assess whether adding proteins to established risk factors improved prediction, we tested the performance of the variables included in the NDR risk calculator with and without the protein values. The NDR model (https://www.ndr.nu/IFrameRisk/) [13] was developed in the Swedish NDR to predict 5 year risk of MACE in 30–75-year-olds with type 2 diabetes and comprises age of onset and duration of type 2 diabetes, log_*e*_(total cholesterol/HDL-cholesterol), log_*e*_(HbA_1c_), log_*e*_(systolic blood pressure), log_*e*_(BMI), sex, current smoker, microalbuminuria, macroalbuminuria, atrial fibrillation and history of cardiovascular disease. This is recommended for evaluating cardiovascular risk in adults with type 2 diabetes by the Swedish National Board of Health and Welfare [26].

We selected the NDR variables for our baseline risk model but used a different statistical approach than that used by Zethelius et al, who developed the NDR calculator [13]. The combined cohorts were randomly split into training (75%) and test (25%) datasets. Cox gradient boosted machine (GBM) learning [27] was applied to the training sample. A baseline model with NDR variables and a baseline-plus-protein model were derived. GBM variables were optimised with regard to model performance (AUC) and complexity as explained in ESM Methods. C statistic, sensitivity and specificity were estimated in the separate test sample. Performance measures and CIs were obtained by bootstrapping in 1000 random iterations. In order to identify a sparse selection of proteins that need to be added to the NDR risk factors to achieve comparable risk discrimination as the whole assay, we used L1-regularised lasso Cox regression. We forced the NDR risk factors into the model by setting the penalty variable in the cv.glmnet function in R to zero, and trained the model by tenfold bootstrapped cross-validation in a random 75% training sample. The optimum sparse model that minimised the prediction error (selected by lambda.min) was evaluated in the separate 25% holdout test sample. Analyses were performed in R software version 3.3.2 (https://www.r-project.org/) using the packages survival, nephro, mice, powerSurvEpi, gbm, glmnet, pROC and ggplot2.

## Results

### Sample characteristics

Figure 1 illustrates the study flow chart, and Table 1 lists the baseline characteristics of all participants. The discovery sample (CARDIPP) recorded 71 MACE events in 708 participants over a mean (±SD) of 7.3 ± 1.8 years (range 0.1–9.65). At a 5% FDR, we estimated 80% power to detect an HR of 1.41 per 1 SD change in protein signal. The replication sample combined participants with type 2 diabetes in ULSAM (*n* = 86; 37 events over 6.8 ± 3.8 years), PIVUS (*n* = 98; 29 events over 8.1 ± 2.9 years), MIVC (*n* = 140; 38 events over 2.9 ± 1.2 years), SAVa-control (*n* = 80; ten events over 4.9 ± 1.6 years) and PADVa (*n* = 99; 26 events over 4.5 ± 2.0 years). The replication set thus included 503 diabetic individuals, 140 of whom experienced a MACE during 5.2 ± 3.1 years (range 0.01–12.83), with 80% power to detect an HR of 1.28 per SD unit of protein. None of the models violated the proportional hazards assumption (*p* > 0.05).

**Table 1:** Sample characteristics

Variable	Cohort					
	CARDIPP	ULSAM	PIVUS	MIVC	SAVa-control	PADVa
Events/total *N*	71/708	37/86	29/98	38/140	10/80	26/99
Follow-up, years	7.3 ± 1.8	6.8 ± 3.8	8.1 ± 2.9	2.9 ± 1.2	4.9 ± 1.6	4.5 ± 2.0
% women	34.2	0	44.9	35.6	25	27.3
Age, years	60.7 ± 3.1	77.5 ± 0.7	70.1 ± 0.1	62.4 ± 8.7	68.8 ± 8.8	68.2 ± 7.3
BMI, kg/m^2^	30.2 ± 4.7	27.5 ± 3.5	29.2 ± 5.4	30.0 ± 5.2	30.0 ± 4.0	28.5 ± 4.0
HbA_1c_, mmol/mol	52.9 ± 11.7	43.9 ± 14.5	NA	62.6 ± 19.1	52.3 ± 13.0	55.3 ± 12.9
HbA_1c_, %	7.0 ± 3.2	6.2 ± 3.5	NA	7.9 ± 3.9	6.9 ± 3.3	7.2 ± 3.3
eGFR, ml min^−1^ [1.73 m]^−2^	78.2 ± 14.2	64.8 ± 14.3	65.1 ± 14.6	19.9 ± 9.3	66.1 ± 19.3	53.3 ± 18.6
Systolic BP, mmHg	136.8 ± 16.5	154.1 ± 18.5	156.7 ± 30.0	157.7 ± 28.8	145.4 ± 21.0	144.9 ± 22.5
Total cholesterol, mmol/l	4.3 ± 0.7	5.2 ± 0.9	5.1 ± 0.9	4.8 ± 1.5	4.5 ± 1.0	4.8 ± 1.0
LDL-cholesterol, mmol/l	2.6 ± 0.8	3.3 ± 0.8	3.0 ± 0.9	2.6 ± 1.2	2.7 ± 0.9	2.4 ± 0.9
HDL-cholesterol, mmol/l	1.3 ± 0.3	1.3 ± 0.3	1.4 ± 0.4	1.1 ± 0.4	1.2 ± 0.3	1.1 ± 0.3
Current smoker, *n* (%)	129 (18.2%)	6 (7.0%)	11 (11.2%)	63 (45.0%)	3 (3.8%)	11 (11.1%)
History of cardiovascular disease, *n* (%)	76 (10.7%)	17 (19.8%)	13 (13.3%)	62 (44.3%)	15 (18.8%)	31 (31.3%)
Antihypertensive medication, *n* (%)	343 (48.4%)	53 (61.6%)	56 (57.1%)	138 (98.6%)	60 (75.0%)	95 (95.6%)
Statin use, *n* (%)	393 (55.5%)	15 (17.4%)	25 (25.5%)	108 (77.1%)	49 (61.3%)	84 (84.8%)

Continuous variables are given as mean ± SD

### Protein biomarkers associated with risk of MACE

**Fig. 2:**
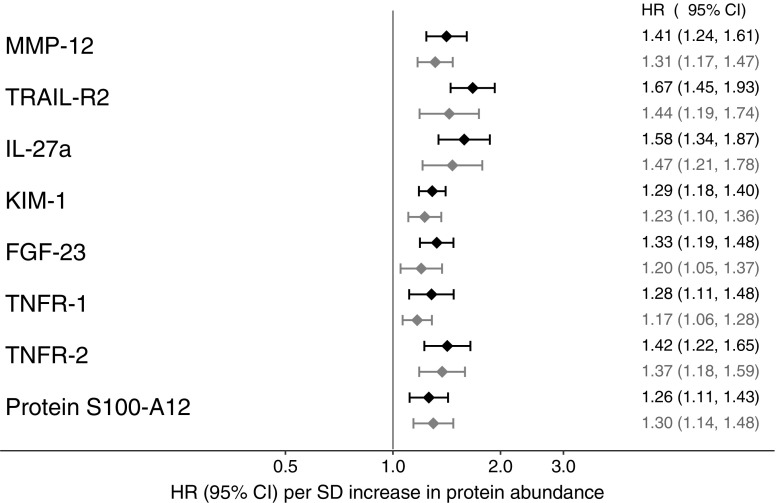
Associations between replicated biomarkers and risk of MACE. Cox regression results in the total sample (*n* = 1211) are given as HR per SD increase in baseline protein levels (error bars denote 95% CIs), and plotted on a log scale. Adjustment for age and sex (black symbols and numbers) is compared with additional adjustment for atrial fibrillation, BMI, HbA_1c_, LDL-cholesterol, microalbuminuria, systolic blood pressure, smoking, statin use, duration of type 2 diabetes, history of cardiovascular disease and eGFR (grey symbols and numbers)

In the discovery sample, 35 out of 80 proteins were associated with prospective risk of MACE at a 5% FDR after adjustment for age and sex (ESM Table 3). Eight associations were replicated at <5% FDR in the separate replication sample (ESM Table 4). In order to test for associations between biomarkers and MACE independent of established risk factors, we combined all cohorts and tested the eight replicated biomarkers in models adjusted for cardiovascular risk factors. Figure 2 shows the results for the eight biomarkers. In the fully adjusted models, increased levels of the following were associated with incident MACE: matrix metalloproteinase (MMP)-12 (HR per SD increase in protein abundance 1.31, 95% CI 1.17, 1.47); TNF-related apoptosis-inducing ligand receptor 2 (TRAIL-R2, also known as death receptor 5) (HR 1.44, 95% CI 1.19, 1.74); IL-27 subunit α (IL-27a; HR 1.47, 95% CI 1.21, 1.78); kidney injury molecule (KIM)-1 (HR 1.23, 95% CI 1.10, 1.36); fibroblast growth factor (FGF)-23 (HR 1.20, 95% CI, 1.05, 1.37); TNF receptor (TNFR)-1 (HR 1.17, 95% CI 1.06, 1.28); TNFR-2 (HR 1.37, 95% CI 1.18, 1.59); and protein S100-A12, also known as extracellular newly identified receptor of advanced glycation end products (RAGE)-binding protein (EN-RAGE; HR 1.30, 95% CI 1.14, 1.48). The exclusion of 12 individuals who had had a haemorrhagic stroke (in MIVC, all five individuals with any type of stroke were excluded as subtypes had not been recorded) had little effect on the effect sizes of the eight biomarkers and replicated the same eight proteins as in the main analysis plus three additional ones (ESM Results, ESM Tables 5–7). A sensitivity analysis with additional adjustment for circulating levels of N-terminal pro-brain natriuretic peptide in those cohorts with available measurements resulted in somewhat increased *p* values, but essentially left the associations between biomarkers and risk of MACE unchanged (ESM Results, ESM Table 8). Correlations between the biomarkers are shown in ESM Table 9.

### Improved risk prediction for MACE in type 2 diabetes

We trained a baseline GBM model including all NDR variables and an NDR-plus-protein model adding all 80 proteins. In the training set of 136 people who experienced a MACE event and 698 people who did not, discrimination improved from C = 0.738 (95% CI 0.735, 0.740) at baseline to C = 0.825 (95% CI 0.824, 0.827) with added proteins (*p* for difference, *p*_diff_ = 3.33 × 10^−54^). Discrimination in the separate test sample of 49 people with and 229 without a MACE event improved from C = 0.686 (95% CI 0.682, 0.689) to C = 0.748 (95% CI 0.746, 0.751; *p*_diff_ = 8.48 × 10^−21^). Sensitivity and specificity in the test sample for the upper 50th risk percentile were 70.9% and 54.0%, respectively, for baseline, and 79.1% and 55.8% with added proteins. Sensitivity and specificity for the upper 25th risk percentile in the test set were 48.1% and 78.9%, respectively, for baseline, and 53.6% and 80.4% for added proteins. Lasso Cox regression in the training sample selected 20 proteins in addition to the NDR risk factors. The prediction performance in the independent test sample had a C statistic of 0.747 (95% CI 0.653, 0.842). A model that included the eight replicated biomarker proteins discovered in part 1 resulted in a C statistic of 0.736 (95% CI 0.641, 0.829).

## Discussion

In this prospective multicohort study of adults with type 2 diabetes, we used multiplex proteomics to identify four novel biomarkers associated with prospective risk of a major cardiovascular event independent of potential confounders. Addition of proteomics data to established risk factors improved the 6 year risk prediction of cardiovascular events.

### Novel biomarkers for cardiovascular risk in diabetes

We identified eight circulating biomarkers, including four novel ones, for incident cardiovascular events after adjustment for established risk factors. Our results replicate previous findings in individuals with type 2 diabetes of associations of increased levels of MMP-12 [17], FGF-23 [28], TNFR-1 and TNFR-2 [29] with incident MACE. For the other four biomarkers, we found no previous studies of prospective associations with MACE in type 2 diabetes, although all have been implicated in cardiometabolic disease in other settings.

Protein S100-A12 (EN-RAGE), the ligand for RAGE, has been associated with incident type 2 diabetes [30] and risk of coronary heart disease [31]. Interaction between RAGE and EN-RAGE triggers an inflammatory cascade, and it has been shown that expression of protein S100-A12 in vascular smooth muscle cells induces oxidative stress, inflammation and vascular remodelling [32].

KIM-1 is mainly expressed in the apical membrane of the renal proximal tubule, and raised circulating levels of KIM-1 are associated with progressive stages of chronic kidney disease in individuals with type 2 diabetes [33, 34]. Associations between raised plasma levels of KIM-1 and adverse cardiovascular risk factors in the general population have recently been reported [35]. Our results in analyses adjusted for kidney function support a potential role of circulating KIM-1 as a cardiovascular risk marker independent of its association with renal function. Our study cannot address the pathogenic mechanisms or potential causality linking KIM-1 to cardiovascular risk in type 2 diabetes, and future experimental studies are indicated.

TRAIL-R2 is a cell surface receptor for TNF-related apoptosis-inducing ligand (TRAIL), involved in apoptosis. Raised circulating TRAIL-R2 levels have been linked with cerebral atherosclerosis [36] and increased mortality in acute myocardial infarction [37]. Possible mechanisms linking the TRAIL/TRAIL-R2 pathway to atherosclerotic disease involve the endothelial response to cholesterol deposits [37, 38] and the composition of circulating fatty acids, as a study in an Alaskan Inuit population found an association between plasma fatty acid levels and genetic variants of the TRAIL-R2 gene *TNFRSF10B* [39].

IL-27 has complex pro- and anti-inflammatory effects that include direct modification of CD4^+^ and CD8^+^ T cells, as well as roles in both innate and antibody-mediated immunity [40]. It has been linked, for instance, to type 1 diabetes [41] and improved atherosclerosis in mice [42], yet functional genetic variants of *IL27* were not associated with cardiovascular outcomes in a sample of Chinese individuals [43]. The roles of the four new biomarkers in inflammatory pathways point to an important role of the immune system in cardiovascular pathology in type 2 diabetes. Whether the novel biomarkers might serve as treatment targets remains to be assessed in future studies.

### Multiplex proteomics improves prediction beyond established risk factors

The addition of proteins to the variables included in the NDR risk model significantly improved cardiovascular risk prediction. In our test sample, added biomarkers improved discrimination from 68.6% to 74.8%, compared with 72.0% reported in the original publication of the NDR model [13]. The model containing the NDR risk factors plus proteins also improved sensitivity and specificity for the upper half (79.1% and 55.8%, compared with 76.2% and 52.9%, respectively, in the original NDR model [13]) and the upper quarter of predicted risk (53.6% and 80.5%, compared with 51.2% and 77.9%, respectively). Importantly, direct comparisons with the NDR calculator are not possible as we used a different statistical method and study design, as well as a smaller test sample and a somewhat longer follow-up of approximately 6 years. The crucial comparisons are therefore the test set performances in our own sample. Predictor selection with lasso regression retained a subset of 20 proteins in addition to risk factors and achieved a near-identical discrimination performance (C = 0.747) as the model including all 80 proteins. Our results demonstrate that adding proteomics data to known risk factors might aid decision-making for cardiovascular prevention in individuals affected by type 2 diabetes. The protein assay used in this study analyses small sample volumes in under 48 h, making it potentially useful for clinical practice. The accessibility of proteomics platforms is likely to increase in the coming years, and a number of studies have demonstrated how proteomics can discover new biological insights [16–18].

Clinical decisions about whether more aggressive cardiovascular prevention with newer drugs will benefit individuals with type 2 diabetes are difficult, given the progressively smaller benefits, risk of side effects and treatment costs [7–9, 11, 14]. In this study, we demonstrate how a multi-biomarker assay can improve risk prediction; future studies in an embedded healthcare setting are indicated to assess the value of ‘-omics’ methods in day-to-day practice. Targeted cardiovascular proteomics might also be useful for streamlining clinical trials of cardiovascular prevention by risk-stratifying participants for cardiovascular prevention, which could lead to improved power to detect clinically meaningful effects and limit expenses [44]. Any application of proteomics with clinical consequences, however, first requires careful validation in future studies.

### Strengths and limitations

Strengths of our study include the prospective community samples, a discovery/replication design and the use of a low sample-volume assay with high-specificity antibody doublets. We limited the risk of overfitting by replicating results in a separate random test subsample and averaging across 1000 iterations, but the bootstrapped CIs have to be interpreted with caution, and our model should be replicated in an independent study. The C statistic of the baseline model was somewhat lower than expected, which may have led to overoptimistic results after adding proteins. On the other hand, the C statistic is usually rather insensitive to added predictors, and we showed convincing improvement [45]. Limitations include the moderate sample size, lack of power to assess the components of MACE as separate outcomes, and failure of 12 proteins in quality control because of missing values. Generalisability is limited to middle-aged to elderly adults (30–77 years of age). Analyses accounted for heterogeneity between cohorts and, rather than limiting variability and effective sample size by tightening the inclusion criteria, we attempted to increase external validity by including a broader range of individuals with type 2 diabetes that would reflect clinical reality.

## Conclusion

We found that a high-throughput multiprotein assay for presumptive disease markers can identify novel biomarkers and improve the identification of individuals with type 2 diabetes at highest risk of a cardiovascular event. Larger clinic-based studies are needed to assess the value of multiplex proteomics in a healthcare context.

## Electronic supplementary material

ESM Tables 1–9 (XLSX 62 kb)

ESM (PDF 68 kb)

## Data Availability

The authors report that, for approved reasons, some access restrictions apply to the data underlying this study. Phenotypes from ULSAM, PIVUS, CARDIPP, MIVC, SAVa-control and PADVa are not publicly available for ethical reasons, as agreed upon by participating volunteers in their informed consent. Data are available on request for researchers who meet the criteria for confidential data access. Data from the ULSAM study are available from the ULSAM steering committee (http://www.pubcare.uu.se/ulsam/Database; contact: V. Giedraitis, vilmantas.giedraitis@pubcare.uu.se). Data from the PIVUS study are available from the PIVUS steering committee (http://www.medsci.uu.se/pivus/; contact: lars.lind@medsci.uu.se). Data from the MIVC study are available from the MIVC steering committee (contact: A. Cordeiro, accordeirojr@uol.com.br). Data requests for the PADVa/SAVa study should be addressed to the steering group (https://savastudy.se/coworkers/; contact: P. Hedberg, par.o.hedberg@regionvastmanland.se). Data requests in the CARDIPP study should be addressed to the steering committee (details: https://clinicaltrials.gov/ct2/show/NCT01049737; contact: C. J. Östgren, carl.johan.ostgren@liu.se).

## Abbreviations

CARDIPP: Cardiovascular Risk Factors in Patients with Diabetes: a Prospective Study in Primary Care
EN-RAGE: Extracellular newly identified RAGE-binding protein
FDR: False discovery rate
FGF: Fibroblast growth factor
GBM: Gradient-boosted machine
KIM: Kidney injury molecule
MACE: Major adverse cardiovascular event/s
MIVC: Malnutrition, Inflammation and Vascular Calcification
MMP: Matrix metalloproteinase
NDR: National Diabetes Register
PADVa: Peripheral Arterial Disease in Västmanland
PIVUS: Prospective Investigation of the Vasculature in Uppsala Seniors
RAGE: Receptor of advanced glycation end products
SAVa: Study of Atherosclerosis in Västmanland
TNFR: TNF receptor
TRAIL: TNF-related apoptosis-inducing ligand
TRAIL-R: TNF-related apoptosis-inducing ligand receptor
ULSAM: Uppsala Longitudinal Study of Adult Men
VaMIS: Västmanland Myocardial Infarction Study
