# Supplementary material for: Multiplex proteomics for prediction of major cardiovascular events in type 2 diabetes
Source: Diabetologia. 2018 May 24;61(8):1748–57. doi: 10.1007/s00125-018-4641-z (PMC6061158; doi:10.1007/s00125-018-4641-z)
Supplement: Supplementary file 2 — (PDF 68 kb) [file 125_2018_4641_MOESM2_ESM.pdf]

Multiplex plasma proteomics for prediction of major cardiovascular events in type 2 diabetes

Nowak et al.

ELECTRONIC SUPPLEMENTARY MATERIAL (ESM)

ELECTRONIC SUPPLEMENTARY MATERIAL (ESM)

ESM Methods

ESM Results

ESM Table 1. Proteins measured by the assay

ESM Table 2. % missing proteins in each cohort.

ESM Table 3. Results of the discovery analysis

ESM Table 4. Results of the replication analysis

ESM Table 5. Results of the discovery analysis after exclusion of cases of hemorrhagic stroke (n = 7, all cohorts other than MIVC) or any stroke (6 cases in MIVC)

ESM Table 6. Results of the replication analysis after exclusion of cases of hemorrhagic stroke (n = 7, all cohorts other than MIVC) or any stroke (6 cases in MIVC)

ESM Table 7. Results for the eight biomarkers in adjusted models in the total sample (also displayed in Figure 1) and after excluded cases of hemorrhagic stroke (n = 7, all cohorts other than MIVC) or any stroke (6 cases in MIVC)

ESM Table 8. Association results after additional adjustment for NT-pro-BNP.

ESM Table 9. Pearson correlation between the eight biomarker proteins in the total sample.

ESM METHODS

Multiplex proteomics

The Olink Proseek Multiplex CVD I 96x96 proximity extension assay [1, 2] quantifies 92 proteins with presumptive roles in cardiovascular or inflammatory disease (listed in **ESM Table 1**) and four internal control samples. It requires ~10 microliter of sample distributed across a 96-well plate. Proteins are targeted by two specific antibodies, each tagged with a single-stranded oligonucleotide sequence. When both antibodies bind their distinct epitopes on the protein, their close spatial proximity allows the partially complementary oligonucleotides to anneal with the aid of added DNA polymerase enzyme. The annealed DNA molecule, which is unique to each protein, then serves as a reporter sequence for quantitative polymerase chain reaction (PCR) amplification. The number of PCR quantification cycles (Cq) required to reach the fluorescence detection threshold is inversely proportional to the protein concentration. Cq values are corrected for technical variation by subtracting sample Cq and interplate-control Cq from the negative control Cq per plate. Resulting $\log_2$-scaled values reflect relative protein abundance but are not readily convertible to concentration values.

The assay uses two specific antibodies for each protein and has been thoroughly assessed for reliability and validity (detailed here http://www.olink.com/wp-content/uploads/2015/12/0696-v1.3-Proseek-Multiplex-CVD-I-Validation-Data_final.pdf).

The lower limit of detection was defined as 3 standard deviations above background noise. Validation of the assay was performed at two separate laboratories with a mean coefficient of validation (CV) of 6-9% intra-assay, 13-17% inter-assay, and 10-16% inter-site. Overall average inter-site variation was 15%. Potential interference from heterophilic antibodies such as rheumatoid factors was excluded by a "mismatch" system as described her http://www.olink.com/wp-content/uploads/2015/12/0696-v1.3-Proseek-Multiplex-CVD-I-Validation-Data_final.pdf. The potential interference from serum and plasma components was ruled out in tests with serial dilutions of bilirubin, hemolysate and lipids. Scalability was ascertained by comparing 24-, 48-, 72- and 96-plex assay run, which produced R2 values >0.99 [1, 3]. In this study, abundance values were normalized within each cohort to mean 0 and standard deviation 1.

Proteins excluded in quality control

All proteins with > 15% missing values in a cohort were excluded resulting in the removal of 12 of the 92 proteins on the assay. All other missing values were imputed as lower limit of detection (LOD) divided by. Twelve proteins were excluded as > 15 % values were missing in at least one cohort. The percentage of missing values per cohort are reported in ESM Table 2.

Gradient boosted machine learning (GBM)

All cohorts were combined at the individual level and randomly split into a 75%-training set and a 25%-validation set. In the training set, a Cox proportional hazards GBM model adjusted for cohort that included all covariates of the Swedish National Diabetes Register risk predictor (onset age and duration of T2D, log(total cholesterol / HDL-cholesterol), log(HbA1c), log(systolic blood pressure), log(BMI), sex, current smoker, microalbuminuria, macroalbuminuria, atrial fibrillation and history of cardiovascular disease) was trained. A second GBM model with the same covariates plus all 80 quality-controlled proteins (scaled to mean 0, SD 1) was also trained. The performance of both models was tested in the separate validation set by comparing C-statistics and sensitivity/specificity of the upper 50% and upper 25% predicted risk. GBM parameters were optimized over multiple iterations in the training sample and set to ≤ 1,000 trees (evaluated parameters: 1,000; 500; and 100), 2-way interaction depth (evaluated 1-; 2- and 3-way interaction), ≥ 5 observations in terminal nodes (evaluated 5; 10; and 20) and shrinkage 0.01 (evaluated 0.01 and 0.001). Permutations of all parameter combinations were compared by area under the curve and the least complex combination beyond which model performance did not improve perceptibly (i.e. area under the curve change by less than 0.1), were chosen.

ESM RESULTS

Exclusion of stroke cases

We repeated the replication / discovery analysis after excluding all cases of hemorrhagic stroke (intracerebral hemorrhage or subarachnoid hemorrhage, ICD-10 codes I60-I62; 5 cases in CARDIPP, 1 case in ULSAM and 1 case in SAVa). In the MIVC cohort, the type of stroke (ischemic or hemorrhagic) had not been recorded and all 5 cases of stroke were excluded. The discovery and replication results in age-, sex- and cohort-adjusted models are shown in ESM Table 5 and 6, respectively. In the discover sample, 33 proteins passed the 5% false discover rate threshold and were taken forward for replication (ESM Table 5). In the

replication sample (n = 496 following exclusion of 7 cases of stroke), eleven proteins were replicated at a 5% false discovery rate (ESM Table 6). These include the eight biomarkers that were replicated in the main analysis (TRAIL-R2, MMP-12, KIM-1, IL-27A, FGF-23, TNFR-1, TNFR-2 and EN-RAGE), as well as grow and differentiation factor 15 (GDF-15), C-X-C- motif chemokine 16 (CXCL-16) and macrophage colony stimulating factor 1 (CSF-1). ESM Table 7 shows association results for the eight protein biomarkers in Cox regression for risk MACE adjusted for cohort, age and sex with and without additional adjustment for established risk factors as explained in the main text. The associations changed very little after excluding stroke cases from the definition of MACE.

Adjustment for NT-pro-BNP

Measurements of N-terminal pro-brain natriuretic peptides were available in CARDIPP (mean 113 ± 223 ng/L), PIVUS (153 ± 209 ng/L), MIVC (324 ± 1055 ng/L), SAVa (204 ± 406 ng/L) and PADVa (404 ± 895 ng/L). In the combined sample, the eight biomarker proteins had the following Pearson correlation coefficients with NT-pro-BNP: r = 0.1688 for MMP-12, r = 0.2074 for TRAIL-R2, r = 0.1650 for TNFR-1, r = 0.1764 for TNFR-2, r = 0.2627 for FGF-23, r = 0.1321 for Protein S100-A12, and r = 0.1986 for KIM-1. We replicated the risk factor-adjusted analysis reported in the main text in this reduced sample with additional adjustment for NT-pro-BNP. The association results with risk of MACE were very similar with and without adjustment for NT-pro-BNP and are presented in ESM Table 8.

REFERENCES

1. Assarsson E, Lundberg M, Holmquist G, et al. (2014) Homogenous 96-plex PEA immunoassay exhibiting high sensitivity, specificity, and excellent scalability. PloS one 9: e95192

2. Lundberg M, Eriksson A, Tran B, Assarsson E, Fredriksson S (2011) Homogeneous antibody-based proximity extension assays provide sensitive and specific detection of low-abundant proteins in human blood. Nucleic acids research 39: e102

3. Enroth S, Johansson A, Enroth SB, Gyllensten U (2014) Strong effects of genetic and lifestyle factors on biomarker variation and use of personalized cutoffs. Nature communications 5: 4684
